# Global Proteotoxicity Caused by Human β_2_ Microglobulin Variants Impairs the Unfolded Protein Response in *C. elegans*

**DOI:** 10.3390/ijms221910752

**Published:** 2021-10-04

**Authors:** Sarah C. Good, Katherine M. Dewison, Sheena E. Radford, Patricija van Oosten-Hawle

**Affiliations:** Faculty of Biological Sciences, School of Molecular and Cell Biology & Astbury Centre for Structural Molecular Biology, University of Leeds, Leeds LS2 9JT, UK; bs12scg@leeds.ac.uk (S.C.G.); bskmd@leeds.ac.uk (K.M.D.); s.e.radford@leeds.ac.uk (S.E.R.)

**Keywords:** β_2_ microglobulin, systemic amyloidosis, protein misfolding, *C. elegans*, extracellular, ER stress, UPR^ER^, proteotoxicity, stress response

## Abstract

Aggregation of β_2_ microglobulin (β_2_m) into amyloid fibrils is associated with systemic amyloidosis, caused by the deposition of amyloid fibrils containing the wild-type protein and its truncated variant, ΔN6 β_2_m, in haemo-dialysed patients. A second form of familial systemic amyloidosis caused by the β_2_m variant, D76N, results in amyloid deposits in the viscera, without renal dysfunction. Although the folding and misfolding mechanisms of β_2_ microglobulin have been widely studied in vitro and in vivo, we lack a comparable understanding of the molecular mechanisms underlying toxicity in a cellular and organismal environment. Here, we established transgenic *C. elegans* lines expressing wild-type (WT) human β_2_m, or the two highly amyloidogenic naturally occurring variants, D76N β_2_m and ΔN6 β_2_m, in the *C. elegans* bodywall muscle. Nematodes expressing the D76N β_2_m and ΔN6 β_2_m variants exhibit increased age-dependent and cell nonautonomous proteotoxicity associated with reduced motility, delayed development and shortened lifespan. Both β_2_m variants cause widespread endogenous protein aggregation contributing to the increased toxicity in aged animals. We show that expression of β_2_m reduces the capacity of *C. elegans* to cope with heat and endoplasmic reticulum (ER) stress, correlating with a deficiency to upregulate BiP/*hsp-4* transcripts in response to ER stress in young adult animals. Interestingly, protein secretion in all β_2_m variants is reduced, despite the presence of the natural signal sequence, suggesting a possible link between organismal β_2_m toxicity and a disrupted ER secretory metabolism.

## 1. Introduction

Amyloid diseases such as Alzheimer’s Disease, Parkinson’s Disease and Dialysis-Related Amyloidosis are characterised by the self-assembly of proteins into insoluble amyloid fibrils containing a cross-β fold that leads to age-dependent cellular toxicity [1,2,3,4]. β_2_ Microglobulin (β_2_m), is a 99 amino acid protein with a β-sandwich immunoglobulin fold in its native state [5,6], that comprises the non-covalently bound light chain of the major histocompatibility complex class I (MHC I) [7]. In patients undergoing long-term haemodialysis, β_2_m dissociates from MHC I and evades dialysis due to its small size, leading to a > 60-fold increase in concentration in the plasma compared with healthy individuals [7]. As a consequence, WT β_2_m self-associates, forming amyloid fibrils that deposit in osteoarticular tissues, causing a pathology known as dialysis-related amyloidosis (DRA) [8]. Amyloid deposits of β_2_m are mainly composed of WT β_2_m (~70%), but also contain truncation products formed by proteolysis, of which 30% represent a six amino acid N-terminal truncation, known as ΔN6 β_2_m [9]. In contrast with WT β_2_m, the ΔN6 β_2_m variant is highly amyloidogenic, and can readily aggregate into amyloid fibrils in vitro at the physiological pH of 6–7, in the absence of additives or fibril seeds [10,11,12]. ΔN6 β_2_m has also been shown to induce amyloid formation of WT β_2_m in vitro [10] and co-assembles with WT β_2_m into amyloid fibrils [13]. In addition, point mutations in β_2_m can increase its aggregation propensity and cause disease, such as the Asp76Asn (D76N) point mutation that underlies a rare hereditary systemic amyloidosis [14]. Patients with D76N β_2_m develop extensive amyloid deposits in visceral tissues that are not a result of dialysis complications, thus showing an aggressive aggregation rate and toxicity of the variant protein.

Although β_2_m is the major component of the amyloid deposits found in DRA, the mechanism by which β_2_m exerts its toxic effects both in vitro and in vivo is less well understood. In vitro, β_2_m fibrils disrupt artificial lipid membranes, [15], whereby fibril-lipid interactions at the fibril ends can result in membrane distortion [16,17]. Moreover, fragmented fibrils are more readily internalised by cells and can accumulate in lysosomes, thereby inhibiting protein degradation by lysosomes and perturbing trafficking via the endo-lysosomal pathway [18,19].

In order to counteract the detrimental consequences of amyloid protein misfolding, cells use an intricate repertoire of defence mechanisms to restore cellular proteostasis and increase organismal survival and healthspan [20]. These mechanisms include cellular stress responses that maintain proteostasis by upregulating a protective chaperone response, such as the HSF-1 mediated heat shock response (HSR) in the cytosol [21] and the unfolded protein response (UPR) of the endoplasmic reticulum (ER) [22]. The accumulation of misfolded proteins in the ER lumen can activate the UPR to initiate processes that adjust its capacity to deal with the increased load of protein aggregates. ER stress is sensed by the inositol-requiring enzyme 1 (IRE-1) that then transduces the activation of the XBP-1s transcription factor to activate UPR genes such as ER-resident chaperones including Grp78/BiP, and ER-associated degradation proteins (ERAD) [23] to restore the challenged protein folding environment in the ER [22]. Importantly, the IRE-1 branch of the UPR is required for protein secretion as well as degradation of misfolded proteins passing through the secretory pathways and into the extracellular environment [24]. Many amyloid pathologies are associated with the accumulation of extracellular amyloid deposits [25,26,27] and the UPR^ER^ is often affected by the accumulation of amyloid proteins destined for secretion, such as transthyretin and amyloid beta (Aβ) peptides [28,29,30]. Via secretion of non-native proteins, the ER can even influence homeostasis in the extracellular environment [25].

Several animal models of amyloidosis have been created, including mice, Drosophila and *C. elegans* [31,32,33,34]. Transgenic *C. elegans* strains expressing either WT β_2_m, the six amino acid truncated β_2_m variant ΔN6, the familial variant of β_2_m, D76N, or the non-naturally occurring variant of β_2_m, P32G, in the body wall muscle, has been shown to affect larval growth, to shorten lifespan, and to reduce motility [31,32,33]. However, the mechanisms of the toxicity observed in these *C. elegans* amyloidosis models were not explored, nor is it understood how β_2_m affects the cellular proteostasis network in an age-dependent manner. Thus, there is a pressing need for the development of animal models of β_2_m-associated amyloidosis in order to understand the cellular and molecular basis of the disease pathology in vivo to help facilitate the development of potential therapies.

Here we generated and characterized a *C. elegans* model of amyloidosis that expresses WT β_2_m, the ΔN6 β_2_m variant or the familial variant, D76N β_2_m. We find that expression of both β_2_m variants in the *C. elegans* bodywall muscle results in cell non-autonomous toxicity in an age-dependent manner, leading to reduced lifespan, fecundity and motility. Underlying these effects is the increased accumulation of endogenous protein aggregation in both variants and the inability of β_2_m-expressing worms to mount an effective UPR^ER^ and cytosolic heat shock response (HSR). The expression of all β_2_m variants decreases the amount of the secreted lipid binding protein LBP-2 into the *C. elegans* pseudocoelom, supporting a model whereby a disrupted UPR^ER^ is linked with a compromised secretory metabolism.

## 2. Results

### 2.1. Generation of C. elegans Models Expressing Human β_2_m Variants

**Figure 1:**
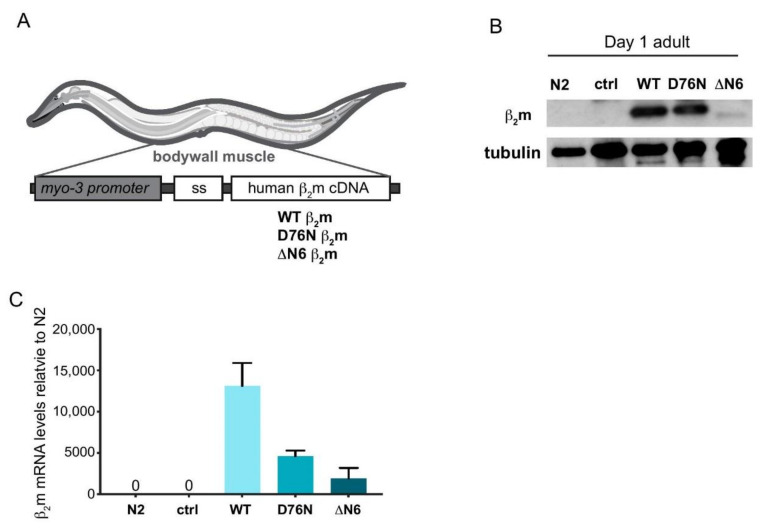
Generation of *C. elegans* β_2_m models. (**A**) β_2_M *C. elegans* transgenes generated in this study. Human β_2_m variants WT, D76N β_2_m and ΔN6 β_2_m with the natural human β_2_m N-terminal signal sequence (SS) were expressed under control of the muscle-specific *myo-3* promoter. (**B**) Representative Western blot analysis of whole nematode extracts of wild-type (N2 Bristol) or transgenic *C. elegans* Day 1 adults expressing *myo-2p::mCherry* (ctrl), WT β_2_m, D76N β_2_m or ΔN6 β_2_m. Immunoblots were probed with anti-β_2_m antibody or anti-tubulin antibody as a loading control. The full (uncropped) version of the Western Blot is shown in Supplementary Figure S1A. (**C**) β_2_m transcript levels in Day 1 adults of N2, *myo-2p::mCherry* (ctrl) and β_2_m expressing transgenic nematodes WT β_2_m, D76N β_2_m or ΔN6 β_2_m. Three independent experiments were performed (*n* = 50 animals per experiment); error bars represent S.E.M.

To model β_2_m proteotoxicity in vivo we expressed human WT β_2_m, and its variants ΔN6 β_2_m and D76N β_2_m in the *C. elegans* bodywall muscle under control of the *myo-3p* promoter (Figure 1A). We generated untagged versions of the three β_2_m transgene variants to avoid known solubility issues of a fluorescent chromophore tag such as GFP [35], as well as potential issues with secretion of the amyloid protein. In each transgenic strain, the human 20 amino acid β_2_m signal sequence (ss) was included to enable β_2_m insertion into the ER and to target it for secretion, to mimic the natural extracellular location of the protein. (Figure 1A).

Expression of β_2_m protein was analysed by Western blot in age-synchronised Day 1 adult nematodes. As shown in Figure 1B, ΔN6 β_2_m protein levels were lower compared with protein levels of the WT and the D76N β_2_m variant (Figure 1B). To investigate whether this is reflected by comparable transcriptional expression levels, we measured β_2_m transcripts by quantitative real time polymerase chain reaction (qRT PCR) (Figure 1C). mRNA levels of the ΔN6 β_2_m variant was 6-fold lower relative to WT β_2_m, (Figure 1C), suggesting the lower ΔN6 β_2_m protein level correlates with a lower level of transcription (Figure 1B).

Human β_2_m WT and the D76N β_2_m variant expressed in *C. elegans* show the same molecular weight as recombinant WT β_2_m which lacks the signal sequence (Figure 1B and Supplementary Figure S1A,B), indicating that the ~2.5 kDa β_2_m signal sequence is cleaved inside the ER, resulting in the expected ~11 kDa molecular weight of the mature β_2_m sequence. For ΔN6, the loss of the N-terminal 6 residues causes a decrease in mass of ~0.72 kDa, which results in a higher mobility on SDS-PAGE Supplementary (Figure S1A,B). Importantly, this protein also migrates with a mobility equivalent to that of the recombinant protein lacking the signal sequence, suggesting complete cleavage of this protein also in vivo to the mature protein sequence.

### 2.2. Expression of D76N β_2_m and ΔN6 β_2_m Variants Delays Development and Reduces Lifespan

To investigate whether the expression of the amyloidogenic β_2_m variants, D76N β_2_m and ΔN6 β_2_m, results in toxic behavioural phenotypes we evaluated characteristic measures representing *C. elegans* health, such as development, brood size and lifespan.

**Figure 2:**
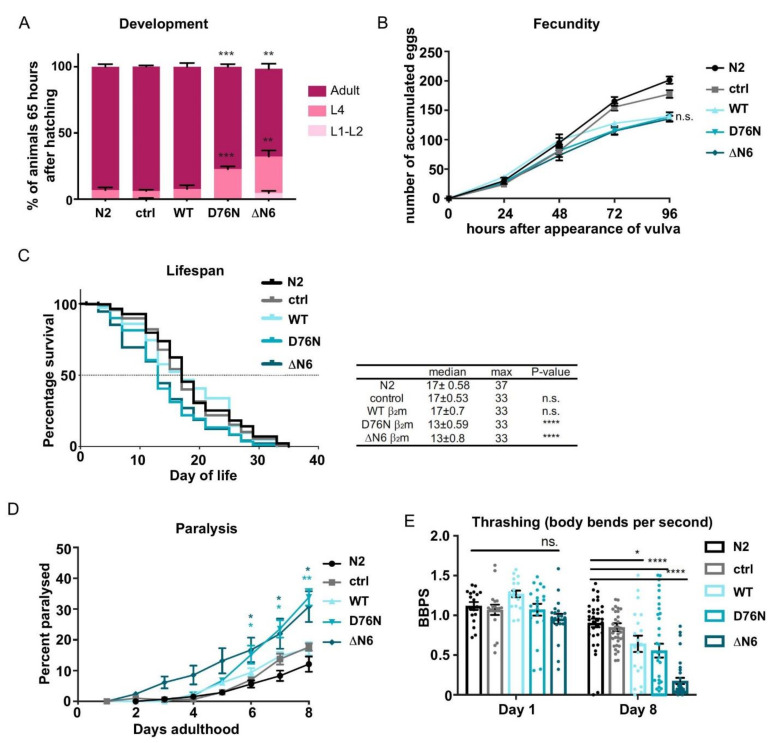
Physiological effects of β_2_m expression on the health of *C. elegans*. (**A**) Development analysis of β_2_m expressing nematodes compared with N2 and control animals (*myo-2p::mCherry*) 65 h after hatching. Data are expressed as the percentage of nematodes on the plate at each developmental stage (L2, L4 and adult stage). Three independent experiments were performed (*n* = 100 animals per experiment). ** *p* < 0.01; *** *p* < 0.001. (**B**) Fecundity assay analysing the number of eggs laid after 24, 48, 72 and 96 h after the appearance of the vulva. Three independent experiments were performed (*n* = 30 animals per experiment). (**C**) Lifespan analysis of WT β_2_m, D76N β_2_m and ΔN6 β_2_m animals compared with control and N2 animals. The plots are representative of three independent experiments with 80-100 nematodes. A log-rank test was performed to calculate statistical significance (**** *p* < 0.0001; n.s. = not significant.). The median and maximum lifespan for each strain is listed in the table right to the graph. (**D**) Paralysis assay of *C. elegans* expressing WT β_2_m, D76N β_2_m and ΔN6 β_2_m compared with wild-type (N2) at 20 °C. The data represent the S.E.M. of three independent experiments (*n* = 100 animals per experiment). * *p* < 0.01; ** *p* < 0.005. (**E**) Thrashing rates of control, WT β_2_m, D76N β_2_m and ΔN6 β_2_m animals compared with N2 at Day 1 and Day 8 of adulthood. Data represent the SEM of the number of full body bends per second (BBPS) in M9. Three independent experiments were performed (*n* = 20 animals). A student’s t test was performed to test significance: ** *p* < 0.01, **** *p* < 0.005; n.s. = not significant.

The majority of animals expressing WT β_2_m or the control strains, N2 Bristol and animals expressing the co-injection marker *myo-2p::mCherry* (named “ctrl”), reach reproductive adulthood 65 h after hatching (Figure 2A). Only a small percentage of N2 and *myo-2p::mCherry* animals (6.8 ± 2.0%) as well as WT β_2_m expressing nematodes (8.4 ± 2.3%) remained at the L4 larval stage at this timepoint. By contrast, the expression of the amyloidogenic β_2_m variants D76N and ΔN6 resulted in a developmental delay with 22.7% ± 2.0 and 32.3% ± 4.6 of animals remaining at the L4 stage, respectively coherent with previous studies [32]. However, we did not observe embryonic lethality of the D76N β_2_m mutant strain as previously reported, which could be due to differences in the signal sequence being used [33] (Figure 2A).

Another measure of animal health is fecundity and we explored whether expression of the amyloidogenic β_2_m variants affected *C. elegans* brood size. For this, we quantified the number of eggs laid from a single hermaphrodite during the first 96 h of adult life at 20 °C. Control animals accumulated an average of 200 eggs within the 96-h time frame (Figure 2B). However, animals expressing WT β_2_m, D76N β_2_m or ΔN6 β_2_m produced 30% less eggs compared to the control, albeit not statistically significant (Figure 2B). Thus, expression of WT β_2_m as well as D76N β_2_m or ΔN6 β_2_m impair *C. elegans* fecundity, while development is only affected by expression of the highly amyloidogenic variants D76N β_2_m and ΔN6 β_2_m.

Finally, we explored how expression of the β_2_m variants influences aging by performing life-span assays (Figure 2C). Wild-type (N2) animals have a median lifespan of 17 ± 0.58 days, and this was unaffected in control strains (17 ± 0.53 days) and *C. elegans* expressing WT β_2_m (17 ± 0.70 days) (Figure 2C). By contrast, a decrease in median lifespan was observed in transgenic animals expressing the amyloidogenic β_2_m variant D76N (13 ± 0.59 days) or the ΔN6 β_2_m variant (13 ± 0.80 days).

Taken together our results show that the highly amyloidogenic β_2_m variants (D76N and ΔN6) increase organismal toxicity by impairing development, fecundity and lifespan, whereas expression of WT β_2_m leads to less severe toxic effects.

### 2.3. Expression of β_2_m Variants in the C. elegans Body Wall Muscle Reduces Motility

We next investigated whether the reduced lifespan of animals expressing D76N β_2_m and ΔN6 β_2_m correlate with reduced healthspan by assessing *C. elegans* motility as a function of age. To address this, the paralysis of transgenic animals was measured for 8 days. At day 8 of adulthood, 17% of WT β_2_m animals were paralysed, comparable with control animals (N2 Bristol) or *ctrl* animals expressing the *myo-2p::mCherry* co-injection marker only (Figure 2D). Nematodes expressing the D76N β_2_m or the ΔN6 β_2_m variant showed a significant increase in paralysis, which was already prevalent at Day 6 of adulthood (15%; *p* < 0.05) and progressed to 34% in D76N β_2_m expressing worms and 31% in ΔN6 β_2_m expressing Day-8 adults (Figure 2D; *p* < 0.01). Interestingly, animals expressing ΔN6 β_2_m, started to display paralysis already at day 3 of adulthood: a much earlier display of a phenotype compared with the D76N β_2_m variant. Thus, the expression of D76N β_2_m and ΔN6 β_2_m is proteotoxic to muscle cells, leading to increased paralysis in *C. elegans* in an age-dependent manner.

To assess the functionality of muscle cells in more detail, we measured the frequency of body bends (thrashing) throughout aging. Control animals N2 Bristol and *myo-2p::mCherry* expressing nematodes (ctrl) displayed an average of 1.12 ± 0.05 body bends per second (BBPS) and 1.07 ± 0.07 BBPS, respectively, at Day 1 of adulthood, and this decreased to 0.90 ± 0.05 BBPS and 0.85 ± 0.06 BBPS, respectively, in Day 8 adults (Figure 2E). WT β_2_m or D76N β_2_m expressing strains performed on average 1.27 ± 0.04 BBPS and 1.07 ± 0.08 BBPS at Day 1 of adulthood, respectively, which was not significantly different to control animals. This thrashing rate then significantly decreased in day 8 adults expressing WT β_2_m (0.65 ± 0.04; *p* < 0.05) or D76N β_2_m (0.56 ± 0.08 BBPS; *p* < 0.0001) (Figure 2E). Expression of ΔN6 β_2_m resulted in slightly (but not statistically significant) decreased thrashing rates in Day-1 adults (0.93 ± 0.10) compared with the control, and this then further decreased to the lowest thrashing rate of all animals (0.18 ± 0.04 BBPS; *p* < 0.0001) at Day 8 of adulthood (Figure 2E). These results indicate that the expression of the β_2_m variants ΔN6 β_2_m and D76N β_2_m, and to a lesser extent, WT β_2_m, in the body wall muscle of *C. elegans* is highly proteotoxic, reflecting the higher aggregation rate of both variants in vitro [36].

### 2.4. Expression of the D76N β_2_m and ΔN6 β_2_m Variants Leads to Wide-Spread Endogenous Protein Aggregation

**Figure 3:**
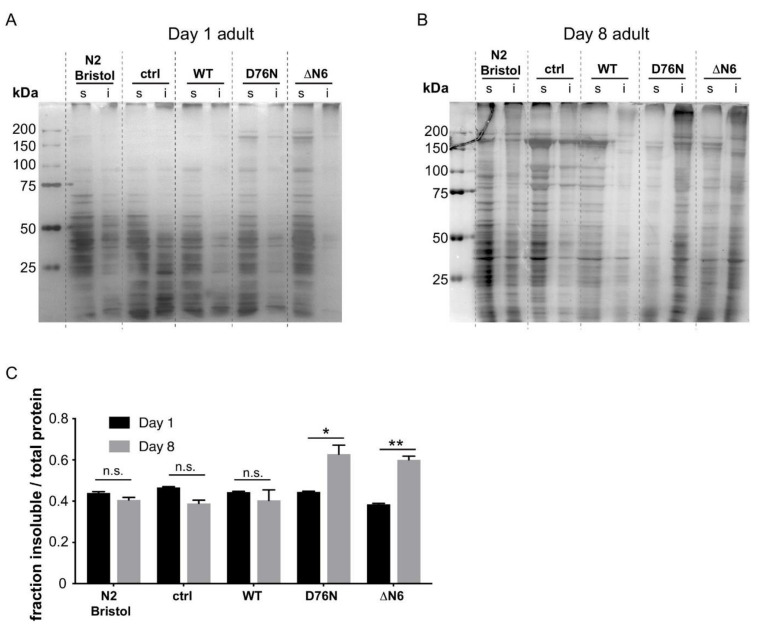
Soluble and insoluble fractions of total protein lysates of Day 1 and Day 8 adult nematodes. Representative silver-stained SDS PAGE of soluble (s) and insoluble (i) fractions of total protein lysates of (**A**) day-1 and (**B**) day-8 adult animals expressing the indicated β_2_m variant. (**C**) Densitometry analysis of the ratio of insoluble protein relative to the total protein (sum of soluble and insoluble) present in Day 1 and Day 8 animals, as shown in SDS PAGE in (**A**) and (**B**). Density of lanes were analysed using ImageJ, and the fraction of insoluble protein was calculated using [density of insoluble proteins]/[density of soluble + density of insoluble proteins]. Western blot images of three independent experiments were analyzed and Student’t t test was used to calculate statistical significance between Day 1 and Day 8. * *p* < 0.05; ** *p* <0.01; n.s. not significant.

To investigate whether the increased organismal toxicity observed for transgenic *C. elegans* expressing the D76N β_2_m and ΔN6 β_2_m variants could be caused by an increased load of misfolded proteins, we assessed the soluble and insoluble fractions of total protein extracts of young (Day 1) and aged (Day 8) adults (Figure 3A,B). Silver staining was used to visualise the total amount of protein present in each sample. In lysates of Day 1 adults, a low proportion of total protein was found in the insoluble fraction in all samples (Figure 3A). By contrast, lysates prepared from Day-8 adults displayed an ~30% higher fraction of insoluble protein in D76N β_2_m and ΔN6 β_2_m samples compared with WT β_2_m (Figure 3B,C). The results demonstrate that the expression of the amyloidogenic β_2_m variants D76N β_2_m and ΔN6 β_2_m cause widespread aggregation of endogenous *C. elegans* proteins in an age-dependent manner. This suggests that the increased aggregation rate of both β_2_m variants disrupts the protein folding environment in vivo, leading to increased global protein aggregation and toxic behavioural phenotypes.

### 2.5. Expression of Amyloidogenic β_2_m Variants Impair Cellular Stress Responses

The proteostasis network (PN) has several stress response pathways to cope with the increased burden of misfolded and aggregated proteins throughout aging and acute environmental stresses [3,21,37]. Because expression of the highly amyloidogenic β_2_m variants D76N β_2_m and ΔN6 β_2_m, increases proteotoxicity, we questioned whether potential failure of the HSR or the UPR could underlie the cytotoxicity caused by expression of the amyloidogenic β_2_m variants.

**Figure 4:**
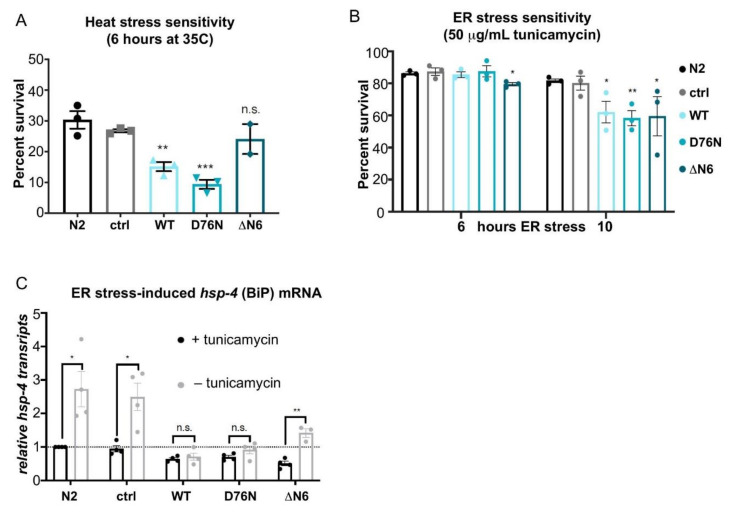
Heat stress and ER stress sensitivity and response. (**A**) Survival rates of N2, control, WT β_2_m, D76N β_2_m and ΔN6 β_2_m Day 1 adults after heat stress. Animals were placed onto seeded NGM plates, incubated in a 35 °C water bath for 6 h, and scored for survival after a 16-h recovery at 20 °C. Three independent experiments were performed (*n* = 50 per experiment). Error bars indicate S.E.M. A Student’s t test was performed to assess significance: ** *p* < 0.005, *** *p* < 0.001, n.s. = not significant. (**B**) ER stress survival rates of N2, control, WT β_2_m, D76N β_2_m and ΔN6 β_2_m Day 1 adults exposed to 50 µg/mL tunicamycin for 6 and 10 h. Survival was scored after 16 h of recovery. Three independent experiments were performed (*n* = 40 animals per experiment). Error bars represent SEM; a Student’s *t* test was performed to calculate significance compared to N2: * *p* < 0.05; ** *p* < 0.01. (**C**) *hsp-4* transcript levels with or without tunicamycin treatment in Day 1 adult control, β2m WT β_2_m, D76N β_2_m and ΔN6 β_2_m animals compared to N2. Animals were incubated in 50 µg/mL tunicamycin for 2 h before RNA was extracted. Three independent experiments were performed (*n* = 50 animals per experiment). Error bars represent S.E.M.; a Student’s *t* test was performed to calculate significance relative to N2: * *p* < 0.05; ** *p* < 0.01; n.s. = not significant.

The ability of animals to cope with heat stress was first investigated. After a 6-h heat shock at 35 °C, *C. elegans* control strains (N2) and *myo-2p::mCherry* expressing animals displayed a survival rate of 30 ± 2.9% and 27 ± 0.5%, respectively (Figure 4A). By comparison, nematodes expressing WT β_2_m or D76N β_2_m were severely affected, with WT β_2_m animals showing a survival rate of only 15 ± 1.5% (*p* < 0.01) and D76N β_2_m expressing animals surviving at a rate of 9 ± 1.5% (*p* < 0.001) (Figure 4A). The expression of ΔN6 β_2_m only slightly reduced *C. elegans* resistance to heat shock, with 24 ± 4.9% of animals surviving the 6-h HS treatment, albeit not statistically significant compared with controls (Figure 4A).

The β_2_m variants expressed in the transgenic *C. elegans* strains are processed in the ER before secretion via the Golgi apparatus. We therefore investigated whether ER stress impairs *C. elegans’* ability to cope with an increased burden of misfolded proteins. To induce ER stress, we treated *C. elegans* with tunicamycin, an inhibitor of N-linked glycosylation that disrupts protein maturation and induces the UPR in the ER [38]. Day 1 adults of control nematodes, N2 and *myo-2p::mCherry* treated with tunicamycin for a 10-h period showed a survival rate of 81.6% (±1.2) and 80.2% (±4.4) respectively (Figure 4B). By contrast, WT β_2_m animals showed a significantly reduced survival rate of 62.1% ± 6.8 (*p* < 0.05) and the D76N β_2_m and ΔN6 β_2_m variants a slightly lower survival rate of 58.4% (±4.7) (*p* < 0.01) and 59.5% (±12.3) (*p* < 0.05), respectively (Figure 4B). Thus, the expression of β_2_m disrupts the organism’s ability to deal with ER stress.

Finally, in order to gain further understanding of this observation, induction of the ER-resident Hsp70 chaperone BiP (*C. elegans hsp-4*) that is upregulated upon ER stress in an IRE-1- and XBP-1- dependent manner was explored [23,39]. Using quantitative RT-PCR, mRNA levels of *hsp-4* were measured during basal conditions and ER stress (Figure 4C). Basal *hsp-4* transcript levels were reduced in all three β_2_m expressing strains (Figure 4C). While ER stress resulted in a 2.7-fold (±0.5) upregulation of *hsp-4* transcript levels in wild-type (N2) animals and a 2.5-fold (±0.4) induction in the *myo-2p::mCherry* control strain, no *hsp-4* induction was measured in WT β_2_m and D76N β_2_m expressing strains (Figure 4C). Interestingly, *hsp-4* transcripts were induced 1.4-fold (±0.1) following ER stress in the ΔN6 β_2_m strain (*p* < 0.01) (Figure 4C) but remained low compared with control strains. Thus, the impaired ability of the β_2_m expressing strains to cope with ER stress is reflected in the reduced induction of *hsp-4* during basal conditions and in response to ER stress. Although animals expressing the ΔN6 β_2_m variant retain the ability to mount an UPR, the magnitude of *hsp-4* induction is significantly lower when compared with control strains (Figure 4C). In summary, these results demonstrate that cellular stress responses such as the UPR^ER^ and the HSR are impaired in young adults, with β_2_m expression likely disrupting ER homeostasis already at physiological conditions.

### 2.6. Expression of β_2_m Variants Impacts Protein Secretion

One of the ER main functions is to serve as an entry point into the secretory pathway. To investigate whether β_2_m is still able to be secreted into the extracellular space with disrupted ER homeostasis, we took advantage of the extracellular proteostasis reporter lipid binding protein-2 (LBP-2) fused to *tagRFP*. LBP-2::tagRFP is secreted from bodywall muscle cells and aggregates in an age-dependent manner in the pseudocoelom [27]. We hypothesised that if the highly amyloidogenic β_2_m variants indeed are secreted, then this would increase the aggregation of LBP-2::tagRFP in the pseudocoelom.

**Figure 5:**
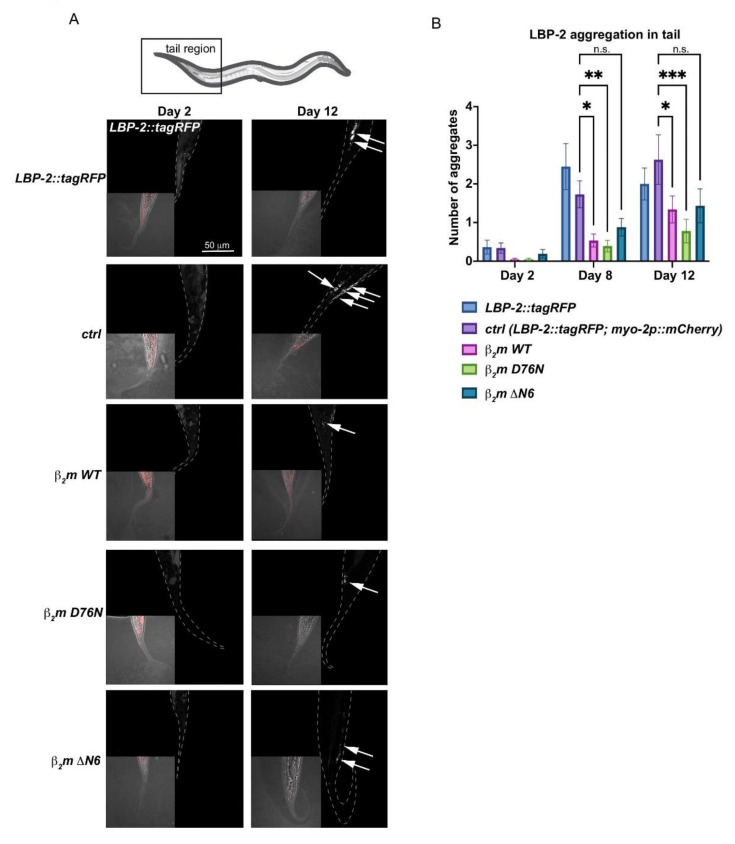
LBP-2 aggregation is reduced in β_2_m expressing nematodes. (**A**) LBP-2::tagRFP puncta in the tail region of day 2 and day 12 adults. Maximum projection is shown. Scale bar, 50 µm. Aggregates are indicated by white arrows. (**B**) Quantification of LBP-2::tagRFP aggregation with age (day 2, day 8, day 12) in β_2_m expressing nematodes compared to control strains. Two independent experiments were performed for each strain (*n* = 10–21 worms per condition per experiment); error bars indicate S.E.M. P-values were determined by a two-way ANOVA followed by a post-hoc Tukey’s test for day 8 and day 12 relative to the ctrl strain (expressing the *myo-2p::mCherry* co-injection marker). * *p* < 0.05; ** *p* < 0.01; *** *p* < 0.001; n.s. = not significant.

In wild-type and control animals expressing the pharyngeal co-injection marker, the number of LBP-2::tagRFP foci in the pseudocoelom increases from an average of ~0.3 foci in Day 2 young adults to an average of ~2.5 aggregates in Day 12 adults (Figure 5A,B), coherent with previous data [27]. Surprisingly, the number of extracellular LBP-2 foci is reduced by 50% in aged WT β_2_m, D76N β_2_m and ΔN6 β_2_m variants compared with controls (Figure 5B). This indicates that protein secretion is less efficient in animals expressing WT β_2_m, D76N β_2_m and ΔN6 β_2_m. As both amyloidogenic variants (D76N and ΔN6) show increased endogenous protein aggregation and all β_2_m-expressing animals have an impaired ER stress response, a possible explanation is that β_2_m protein misfolds in the ER lumen in an age-dependent manner, before it is secreted. This disrupts basic ER functions including degradation of misfolded proteins and impairs protein secretion of LBP-2, similar to *ire-1* and *xbp-1* variants of *C. elegans* that show a defective secretory protein metabolism [24].

## 3. Discussion

In this study we provide a comparison of transgenic *C. elegans* expressing human WT β_2_m with the familial variants D76N β_2_m and ΔN6 β_2_m, all of which are involved in β_2_m-associated amyloid pathology [4,7,8,40]. Consistent with previously generated *C. elegans* β_2_m models [32,33], our strains (which each contain the natural human signal sequence) show increased proteotoxicity at an organismal level in an age-dependent manner. The novelty of our work is that it provides molecular insight into the factors contributing to increased proteotoxicity, which was largely unstudied previously. We show that the highly amyloidogenic β_2_m variants D76N β_2_m and ΔN6 β_2_m cause global aggregation of endogenous *C. elegans* proteins at an advanced age, correlating with the increased cytotoxicity of these variants. Albeit ΔN6 β_2_m expression levels are low in young adults, the insoluble protein fraction of ΔN6 β_2_m expressing nematodes at old age (Day 8) is comparable to that in D76N β_2_m mutants. This suggests that despite the lower expression levels of ΔN6 β_2_m this variant may cause increased aggregation of endogenous proteins in vivo, leading to the observed age-associated proteotoxicity (reduced lifespan, paralysis) that is comparable with the D76N mutant. Thus, even though D76N shows a faster aggregation rate in vitro its proteotoxicity may be superseded by the ΔN6 variant in vivo. Interestingly, all β_2_m variants impair the capacity of the proteostasis network by affecting the animals’ ability to cope with stress conditions. The disrupted ER homeostasis in all β_2_m-expressing nematodes may be linked to reduced secretion of LBP-2, suggesting that both highly aggregation-prone β_2_m variants misfold intracellularly, possibly even inside the ER lumen, thereby impairing ER secretory-protein metabolism in *C. elegans*.

Similar to other *C. elegans* β_2_m models previously established, our models recapitulate reduced lifespan, motility and developmental delays. A previously generated *C. elegans* model expressing D76N β_2_m by Faravelli et al. displayed embryonic lethality that was overcome by the inducible *smg-1* system [33]. While expression of the D76N β_2_m and ΔN6 β_2_m variants in our study caused developmental delays, we did not observe increased embryonic lethality associated with either variant. A potential reason for this could be differences in the expression levels of D76N β_2_m, as the here generated strain expresses D76N β_2_m from an extrachromosomal rather than an integrated array in a *smg-1* mutant strain that requires increased temperature conditions to induce expression of the transgene. Moreover, Faravelli et al., used the endogenous *sel-1* peptide as a secretion signal, whereas our generated version contains the natural human β_2_m signal peptide, which could lead to different secretion ratios.

The accumulation of insoluble endogenous protein deposits combined with a debilitated UPR^ER^ already in young animals, indicates an early impairment of the proteostasis network by the D76N β_2_m and ΔN6 β_2_m variants. While all β_2_m expressing nematodes (WT, D76N and ΔN6) are sensitive to ER stress, it is interesting to note that the heat stress resistance of ΔN6 β_2_m mutants is comparable to control animals, whereas both WT β_2_m and D76N β_2_m are sensitive to heat stress. A possible reason for this discrepancy could be that all three β_2_m variants are targeted to the ER, where they misfold, thereby more severely affecting ER proteostasis. While ΔN6 in particular may be more severely affecting ER proteostasis, it should be noted that expression levels of this variant are also very low. Hence a much lower fraction of misfolded ΔN6 may reach the cytosol where it could impact on cytosolic proteostasis that is challenged by heat stress.

Interestingly, all β2m variants are impaired in their ability to induce *hsp-4* in response to ER stress. Although the ΔN6 mutant shows a slight induction of *hsp-4*, the magnitude of induction is severely reduced and does not reach the level necessary to protect against ER stress as it does in the control animals. This inability to induce a protective ER stress response by upregulation of *hsp-4*/BiP, could be due to an increased oligomeric fraction as was previously reported for ΔN6 β_2_m [32] and D76N β_2_m [33].

In future studies, it will be interesting to investigate whether β_2_m expression in these previously established models exhibit a defective UPR^ER^ as well. Based on our observations suggesting reduced secretion in all β_2_m variants, it is possible that β_2_m oligomers already form in the ER lumen, thereby affecting both the UPR^ER^ and ER-associated degradation (ERAD) before they are secreted. Further work will be required to obtain direct evidence for secretion of β_2_m oligomers/aggregates and/or accumulation of β_2_m in the extracellular environment, as staining of amyloid deposits in the animal by antibody staining or the use of amyloid-reactive dyes such as X-34 or ThT proved inconclusive (data not shown). However, we provide clear evidence that accumulation of LBP-2 in the pseudocoelom decreases with age in all variants consistent with reduced protein secretion, which is a known characteristic for UPR^ER^ pathway variants of the IRE-1 branch [24]. In this respect, it is however important to note differences in the secretion of β_2_m in *C. elegans* compared to human cells. In human cells β_2_m is secreted as part of MHC I before it dissociates to form amyloid species in the extracellular space [41,42]. *C. elegans* does not possess MHC I [43], and directly expresses amyloidogenic forms of β_2_m in these strains. Thus, it is likely that in *C. elegans* proteostasis is first affected intracellularly, before β_2_m oligomeric species are secreted. Such a scenario would explain the reduced capability of β_2_m-expressing animals to induce the UPR^ER^ even at basal conditions. Moreover, our data shows that the signal sequence of ΔN6 β_2_m is successfully removed in the ER lumen in our *C. elegans* model, as the MW of the protein on Western blot is identical to that of recombinant protein (Figure 1B and Supplementary Figure S1A,B). Our data suggests that ΔN6 already misfolds in the ER, as indicated by the increased toxicity and sensitivity to ER stress and reduced lifespan of the mutant strain.

Despite the above limitations of *C. elegans* as a model for β_2_m-associated amyloidosis since it is devoid of MHC I, it is nevertheless a good system to study molecular and organismal aspects of β_2_m amyloidosis. For example, *C. elegans* harbours organs and tissues affected by D76N β_2_m amyloidosis pathology, such as the intestinal tract and muscle tissue of the pharynx, the pumping function of which is often compared to the human heart. This can allow comparisons to the human pathology where the wide-spread concentration of D76N β_2_m amyloid deposits into heart or the gastro-intestinal tracts leads to life-threatening complication [14,44]. Moreover, collagen as well as glycosaminoglycans which are both important drivers of β_2_m amyloid aggregation [12,45,46,47] are highly present in *C. elegans* muscle tissues [48].

In summary, the transgenic *C. elegans* expressing β_2_m variants generated here and in previous studies [32,33] will prove important model systems to gain a robust molecular insight into oligomer formation and understanding of the molecular causes of proteotoxicity in vivo. Our work highlighted the importance of the proteostasis network and a disrupted ER homeostasis combined with increased endogenous protein aggregation as drivers of β_2_m associated toxicity in vivo. A focus in future studies on proteostasis network mechanisms and secretory protein metabolism will further increase our understanding of these diseases in vivo and in the quest of finding potential therapeutic avenues.

## 4. Material and Methods

### 4.1. C. elegans Strains and Maintenance

Wild type (N2 Bristol) and transgenic animals were handled using standard methods [49]. Unless otherwise stated, strains were maintained at 20 °C on 60 mm NGM agar plates seeded with OP50-1 *Escherichia coli* as a food source, according to [49].

#### 4.1.1. Strains Generated in This Study:

**PVH69** pccEx007[*myo3::WT hβ_2_m::GFP::unc-54 3′UTR + myo2::RFP*];
**PVH177** pccEx009[*myo-3p::WT hβ_2_m::unc-54 3′UTR + myo-2p::RFP*];
**PVH178** pccEx010[*myo-3p::D76N β_2_m::unc-54 3′UTR + myo-2p::RFP*];
**PVH179** pccEx011[*myo-3p::ΔN6 β_2_m::unc-54 3′UTR + myo-2p::RFP*];
**PVH182** pccEx024[*myo-2p::RFP*].

#### 4.1.2. Cloning and Generation of Transgenic *C. elegans* Strains

*C. elegans* strains expressing β_2_m in the bodywall muscle under control of the *myo-3p* promoter were constructed in the following manner: The expression vector pPD95_75 (Fire lab, Addgene, Watertown, MA, USA) was digested with AgeI and EcoRI, blunt-ended and re-ligated to generate an expression vector without the GFP cDNA. The 1500 bp *myo-3p* body-wall muscle promoter (synthesized by Eurofins, Wolverhampton, United Kingdom) was ligated into the pPD95_75 vector using HindII and SalI sites. The signal sequence for human WT β2m was generated by oligonucleotide annealing of *SalI-signalpeptideB2m-for* and *BamHI-signalpeptideB2m-rev*. Oligos were mixed in a 1:1 ratio in 10 μL, then placed at 94 °C and allowed to cool until 30 °C. Serial dilutions of the oligos were made (1:5, 1:10, 1:50, 1:100). 5 μL of the undiluted oligos and the serial dilutions were ligated into the successfully sequenced *myo-3p-pPD95_75* vector. Ligations were transformed into DH5α cells. Colony restriction digestion was performed using BamHI and SalI restriction enzymes and analysed for a 100 bp band, successful constructs were sent for sequencing. Finally, human WT, D76N β_2_m and ΔN6 β_2_m cDNA (Radford lab) were PCR amplified using primers *BamHI-B2m-for* and *MscI-B2m-rev* to contain BamHI and MscI restriction sites and ligated into the *myo-3p-ss-pPD95_75* vector. Successful colonies containing a 367 bp β2m band were sent for sequencing.

### 4.2. Primers Used

*BamHI-B2m-for*
5′ AAAGGATCCATTCAAAGAACTCCAAAAATTC 3′
*MscI-B2m-rev*
5′ AAATGGCCATTACATGTCTCGATCCCACTTA 3′
*SalI-signalpeptide B2m-for*
5′ TCGACAAAAATGTCTCGCTCCGTGGCCTTAGCTGTGGCCTTAGCTGTGCTC GCGCTACTCTCTCTTTCTGGCCTGGAGGCTG 3′
*BamHI-signalpeptide B2m-rev*
5′ GATCCAGCCTCCAGGCCAGAAAGAGAGAGTAGCGCGAGCACAGCTAAG GCCACGGAGCGAGACATTTTTG 3′

The transgenes were microinjected into the gonads of the N2 Bristol *C. elegans* strain, using a DNA solution containing either the co-injection marker only (5 ng/µL; *myo-2p::mCherry* (pCFJ90; Addgene)), or in combination with 25 ng/µL of the β2m construct. Multiple extrachromosomal lines were generated, based on the red fluorescent pharyngeal co-injection marker. The established transmitting lines used in this study have ~70% meiotic stability.

### 4.3. Western Blot Analysis and SDS-PAGE Silver Staining

For Western blot analysis, nematodes were collected and lysed on ice using a motorised pestle in lysis buffer (150 mM NaCl, 50 mM Tris pH 7.4, 1 mM EDTA, 0.1% (*v*/*v*) NP-40). Extracts were centrifuged at 13,000 rpm for 1 min to pellet the carcasses and the supernatant collected. The protein extract was run on a 10% (*w*/*v*) Tris-Tricine gel and proteins were electro-transferred to 0.22 μM nitrocellulose membranes. The blots were blocked using 5% (*w*/*v*) milk overnight, washed four times in PBS/Tween (0.1%) for 5 min, then probed with primary anti-human β_2_m rabbit polyclonal antibody (Dako, Santa Clara, CA, USA) at a 1:1000 (*v*/*v*) dilution in PBS/Tween (0.1%) and 1% (*v*/*v*) milk for 2 h. Blots were washed then probed with 1:10,000 (*v*/*v*) dilution HRP-conjugated goat anti-rabbit secondary antibody for 1 h. Blots were developed using ECL (ThermoFisher Scientific, Waltham, MA, USA).

For silver staining of SDS-PAGE, nematode extracts were loaded on an 4–20% (*w*/*v*) SDS-PAGE gel (BioRad, Hercules, CA, USA). The gel was fixed in solution A (50% (*v*/*v*) ethanol, 10% (*v*/*v*) acetic acid) for 30 min, then incubated in solution B (5% (*v*/*v*) ethanol, 1% (*v*/*v*) acetic acid) for 15 min on a gentle rocker. The gel was washed in dH_2_O three times for 5 min, and then incubated in solution D (0.02 g sodium thiosulphate in 100 mL dH_2_O) for 2 min while gently rocking. The gel was washed again in dH_2_O three times for 30 s, then incubated in solution E (0.2 g silver nitrate, 75 μL formaldehyde in 100 mL ddH_2_O) for 20 min. After three washes in dH_2_O for 20 s, the gel was developed in solution F (6 g sodium carbonate, 50 μL formaldehyde, 0.2 mL solution C in 100 mL dH_2_O) until protein bands could be visualised and the reaction was stopped using solution G (5% (*v*/*v*) acetic acid). The gel was then stored in dH_2_O and imaged.

### 4.4. Soluble/Insoluble Protein Fractionation

Animals were aged to day 1 or day 8 adults using NGM containing 50 µg/uL FUdR plates. Nematodes were collected using M9 buffer, and resuspended in lysis buffer (20 mM Tris, pH 7.5, 10 mM β-mercaptoethanol, 0.5% (*v*/*v*) Triton X-100, complete protease inhibitor (Roche, Basel, Switzerland)). Extracts were shock frozen in liquid nitrogen, and the frozen nematode pellet was ground using a motorized pestle, then lysed on ice in the presence of 0.0025 U/mL benzonase (Merck SA, Darmstadt, Germany). Lysates were centrifuged at 1000 rpm for 1 min in a tabletop centrifuge and the supernatant was extracted. 2% (*v*/*v*) N-Lauroylsarcosine was added to the supernatant and was ultracentrifuged at 100,000 *g* for 1 h at 4 °C. The supernatant was removed, and the resulting pellet was resuspended in the same volume of dH_2_O as the supernatant.

### 4.5. Developmental Delay Assay

Nematodes for the assay were synchronised by placing ten gravid adult nematodes on an NGM plate with OP50-1 as a food source and allowed to lay eggs for 2 h before removing. Eggs were left to develop for 65 h and the developmental stage was monitored and recorded. Three independent experiments were performed for each strain (*n* > 200 worms per experiment

### 4.6. Fecundity Assay

Thirty L4-stage nematodes were transferred onto separate individual NGM plates smented with OP50-1. At time-points of 24-, 48-, 72- and 96- hours, individual animals were transferred onto a new NGM plate using a sterilised platinum wire, and the number of eggs laid over the previous 24 h was counted and recorded. Three independent experiments were performed for each strain (*n* = 30 worms per experiment).

### 4.7. Lifespan Assay

Animals were synchronised by egg-laying, and 80–100 L4-stage animals were selected per strain and transferred onto five NGM plates with 20 animals per plate. Every other day each nematode was assessed for survival, death or censorship and numbers were recorded. Animals were assessed as dead if they did not display movement when gently touched on the nose using a platinum wire, and no pharyngeal pumping could be observed. Animals were censored if they crawled up the edge of the plate and became desiccated, burrowed into the agar, displayed the ‘bag of worms’ phenotype where eggs hatched internally, or if they could not be found. Any dead or censored animals were recorded and left on plates, while alive animals were transferred onto a new NGM plate. Data was analysed using the OASIS 2 online software [50]

### 4.8. Paralysis Assay

Animals were synchronised using egg-laying methods, and 100 L4-stage nematodes were transferred onto five NGM plates with OP50-1 (20 animals per plate). Every 24 h for 8 days, each nematode was assessed by the touch-nose response for paralysis and the numbers of mobile, paralysed or censored animals were recorded. Mobile nematodes were determined if they were able to move freely, either when touched by a platinum wire, or when unprompted. Paralysed animals were determined if little movement was seen when touched by a platinum wire, but still displayed minimal movement or pharyngeal pumping to indicate they were still alive. Censored worms included worms that had died, displayed the ‘bag of worms’ phenotype, crawled up the side of the plate, or could not be detected. Mobile worms were moved onto a new plate each day to ensure any progeny did not influence the results. Three independent experiments were performed for each strain (*n* = 100 worms per experiment).

### 4.9. Thrashing Assay

Animals were synchronised by egg-laying and aged to either day 1 or day 8 of adulthood by picking. At the appropriate time point, 15–20 nematodes of each strain were transferred into a 24-well plate containing 1 mL M9 buffer. Animals were left to recover for 1 min, then a video was recorded for 30 s using the ToupCam digital camera (GT vision, Sudbury, United Kingdom). At least three videos of each strain were recorded. Videos were analysed in ImageJ using the WrmTrck plugin [51] to quantify the number of body bends per second (BBPS). Each experiment consisted of 15–20 worms being recorded three times and average speed of recorded movies was considered one biological replicate. Three biological replicates were collected for each strain.

### 4.10. Thermotolerance

Progeny were synchronised by egg-laying and 50 L4-stage animals were selected and placed onto an NGM plate. The next day (day 1 adults), plates were incubated at 35 °C for 6 h then moved immediately into a 20 °C incubator. Animals were left to recover for 16 h at 20 °C, then survival of animals was scored. Animals were scored as alive if movement or pharyngeal pumping was observed. The percentage of alive and dead animals were scored and mean survival rates were determined using Student’s t test. Three independent experiments were performed for each strain (*n* = 50 worms) and error bars indicate S.E.M.

### 4.11. ER Stress Assay

Nematodes were synchronised by egg-laying and L4-stage animals were selected and placed onto new NGM plates. Nematodes were left overnight to grow and 50 day-1 adult worms were transferred using a sterilised platinum wire into M9 buffer containing 50 μg/mL tunicamycin. Animals were incubated in tunicamycin for 6 h at 20 °C on a rotator. After incubation, nematodes were washed three times in M9 buffer and plated onto NGM plates. After 16 h of recovery, animals were scored for survival. Alive animals were determined by their ability to move spontaneously or when stimulated by touch, or pharyngeal pumping was observed. Dead animals were determined through lack of movement or pharyngeal pumping. Percentages of alive and dead animals were calculated and plotted. Three independent experiments were performed for each strain (*n* = 50 worms per experiment); error bars indicate S.E.M.

### 4.12. Quantitative RT-PCR

RNA was extracted using TriZOL reagent and grinding the frozen worm pellet using a pellet grinder on ice 5 × for 30 s. RNA was purified using the Zymo-prep RNA Mini Isolation kit (Zymo Research, Cambridge Biosciences, Cambridge, United Kingdom), and qRT-PCR was performed as described previously [52,53].

### 4.13. Quantification of Extracellular Aggregation Using LBP-2::tagRFP as a Reporter

To quantify LBP-2::tagRFP aggregation in the tail region of *C. elegans*, age-synchronised nematodes at indicated time points were immobilised in 10 mM sodium azide on 2% agarose (*w*/*v*) pads on a microscope slide. Images were captured on a LSM880 upright laser scanning confocal microscope (Zeiss, Oberkochen, Germany) with a 40 × oil objective, using the Zen software. tagRFP was detected using a 561 nm excitation laser and an emission range from 565–650 nm. Representative confocal images are displayed as maximum z-stack projections.

For aggregation quantification, the tail region was defined as the region from the tip of the tail to the anal sphincter. We chose the tail region because of interference of the *myo-2p::mCherry* co-injection marker in the head region of β_2_m and ctrl animals. Aggregates were quantified in a semi-automated process. ImageJ was used to subtract the background and apply a threshold to a maximum intensity projection images. Aggregates were defined as fluorescent puncta when larger than 1 µm^2^. Two independent experiments were performed for each strain (*n* = 10–21 worms per condition per experiment) and error bars indicate S.E.M. For statistical significance, a two-way ANOVA followed by a post-hoc Tukey’s test was performed.

## Supplementary Materials

The following are available online at https://www.mdpi.com/article/10.3390/ijms221910752/s1.
