# Supplementary figures and images for: Global Proteotoxicity Caused by Human β2 Microglobulin Variants Impairs the Unfolded Protein Response in C. elegans
Source: Int J Mol Sci. 2021 Oct 4;22(19):10752. doi: 10.3390/ijms221910752 (PMC8509642; doi:10.3390/ijms221910752)

Supplementary Figure S1

A

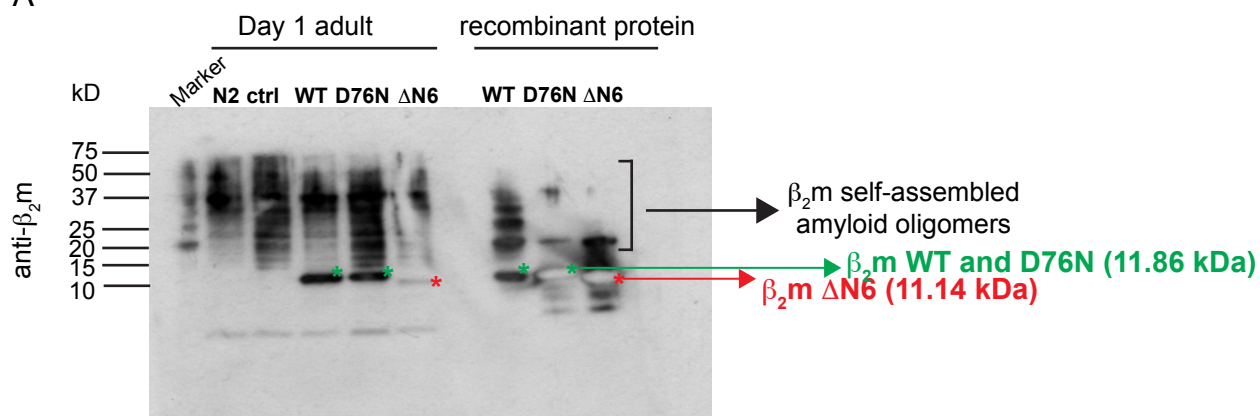

B

Coomassie stained SDS-PAGE of recombinant  $\beta_2$ m protein

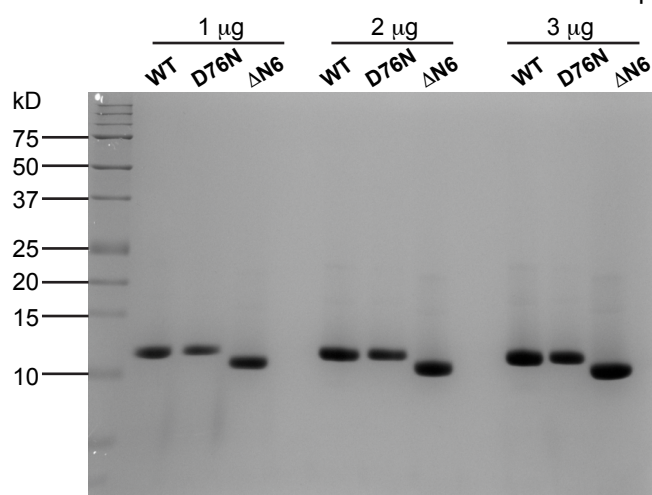

Supplement: Supplementary file 1 [file ijms-22-10752-s001.zip › ijms-1344296-SM- update.pdf]
